# RETRACTED: Fusco et al. Mucosa-Associated Lymphoid Tissue Lymphoma Translocation 1 Inhibitor as a Novel Therapeutic Tool for Lung Injury. *Int. J. Mol. Sci.* 2020, *21*, 7761

**DOI:** 10.3390/ijms27072963

**Published:** 2026-03-25

**Authors:** Roberta Fusco, Rosalba Siracusa, Ramona D’Amico, Marika Cordaro, Tiziana Genovese, Enrico Gugliandolo, Alessio Filippo Peritore, Rosalia Crupi, Rosanna Di Paola, Salvatore Cuzzocrea, Daniela Impellizzeri

**Affiliations:** 1 Department of Chemical, Biological, Pharmaceutical and Environmental Sciences, University of Messina, 98166 Messina, Italyrsiracusa@unime.it (R.S.); rdamico@unime.it (R.D.); tgenovese@unime.it (T.G.); egugliandolo@unime.it (E.G.); aperitore@unime.it (A.F.P.); dimpellizzeri@unime.it (D.I.); 2 Department of Biomedical, Dental and Morphological and Functional Imaging, University of Messina, Via Consolare Valeria, 98125 Messina, Italy; cordarom@unime.it; 3 Department of Veterinary Sciences, University of Messina, 98168 Messina, Italy; rcrupi@unime.it; 4 Department of Pharmacological and Physiological Science, Saint Louis University School of Medicine, Saint Louis, MO 63104, USA

The journal retracts the article “Mucosa-Associated Lymphoid Tissue Lymphoma Translocation 1 Inhibitor as a Novel Therapeutic Tool for Lung Injury” [1] cited above.

Following publication, concerns were brought to the attention of the Editorial Office regarding the integrity of images presented in this publication [1].

Adhering to our standard procedure, an investigation was conducted by the Editorial Office and Editorial Board that identified indications of inappropriate editing and partial duplication between micrographs in Figure 1C,D in this article [1]. While the authors collaborated within this process, raw material meeting the journals requirements for original images could not be provided for Editorial Board Evaluation. Consequently, the Editorial Board has lost confidence in the reliability of the findings and has decided to retract this publication, as per MDPI’s retraction policy (https://www.mdpi.com/ethics#_bookmark30, accessed on 9 March 2026).

This retraction was approved by the Editor-in-Chief of the *International Journal of Molecular Sciences*.

The authors disagreed with this retraction.
